# Cost-Utility and Cost-Effectiveness of Internet-Based Treatment for Adults With Depressive Symptoms: Randomized Trial

**DOI:** 10.2196/jmir.1436

**Published:** 2010-12-19

**Authors:** Lisanne Warmerdam, Filip Smit, Annemieke van Straten, Heleen Riper, Pim Cuijpers

**Affiliations:** ^3^GGZ inGeestMAmsterdamNetherlands; ^2^Trimbos InstituteNetherlands Institute of Mental Health and AddictionUtrechtNetherlands; ^1^Department of Clinical Psychology and EMGO Institute for Health and Care ResearchVU University Medical CentreAmsterdamNetherlands

**Keywords:** Costs and cost analysis, cost-benefit analysis, depression, Internet, computer-assisted instruction, cognitive therapy, problem solving, randomized controlled trial

## Abstract

**Background:**

The effectiveness of Internet-based treatments for depression has been demonstrated; their cost-effectiveness, however, has been less well researched.

**Objective:**

Evaluating the relative cost-utility and cost-effectiveness of (1) Internet-based cognitive behavioral therapy, (2) Internet-based problem-solving therapy, and (3) a waiting list for adults with depressive symptoms.

**Methods:**

A total of 263 participants with clinically significant depressive symptoms were randomized to Internet-based cognitive behavioral therapy (n = 88), Internet-based problem-solving therapy (n = 88), and a waiting list (n = 87). End points were evaluated at the 12-week follow-up.

**Results:**

Cost-utility analysis showed that cognitive behavioral therapy and problem-solving therapy had a 52% and 61% probability respectively of being more acceptable than waiting when the willingness to pay is € 30,000 for one quality-adjusted life-year. When society is prepared to pay € 10,000 for a clinically significant change from depression, the probabilities of cognitive behavioral therapy and problem-solving therapy being more acceptable than waiting are 91% and 89%, respectively. Comparing both Internet-based treatments showed no clear preference for one or the other of the treatments.

**Conclusions:**

Both Internet-based treatments have a high probability of being cost-effective with a modest value placed on clinically significant change in depressive symptoms.

**Trial Registration:**

ISRCTN16823487; http://www.controlled-trials.com/ISRCTN16823487 (Archived by WebCite at http://www.webcitation.org/5u8slzhDE)

## Introduction

Globally, depression is one of the most prevalent mental disorders, with a large impact on peoples’ well-being and with substantial economic ramifications [1-3]. Some cost-effective interventions exist for treating and preventing depression [4-9]. However, economic evaluations of Internet-based treatments are scant [10]. As far as we know, only one economic evaluation has been conducted of Internet-based treatment for depression, showing that online cognitive behavioral therapy can be cost-effective compared with usual care in primary care patients [11]. Moreover, as most Internet-based treatments are based on cognitive behavioral therapy [12], economic evaluation of other forms of Internet-based treatments is also called for.

In a randomized controlled trial for adults with depressive symptoms, Internet-based cognitive behavioral therapy and Internet-based problem-solving therapy showed superior effectiveness compared with a waiting list control group in reducing symptoms of depression and enhancing quality of life [13]. Using the same data, we now investigate the cost-utility and cost-effectiveness of Internet-based treatment. Characteristic of problem-solving therapy in this study is its short duration of only five weeks. It would, therefore, be interesting to know whether this brief intervention is superior to an 8-week cognitive behavioral therapy intervention in terms of relative cost-effectiveness. To that end, we evaluated cost-utility and cost-effectiveness for 3 contrasts: (1) cognitive behavioral therapy versus placement on a waiting list, (2) problem-solving therapy versus placement on a waiting list, and (3) problem-solving therapy versus cognitive behavioral therapy.

## Methods

Full details of the study method are given elsewhere [14]. Here, we describe its main features and focus on the economic aspects.

### Participants

Participants were recruited through advertisements in newspapers and via the Internet. To be included in the study, participants had to be aged 18 years or older, presenting with depressive symptoms, and willing to participate in a self-help course. Presence of depressive symptoms was ascertained with the Center of Epidemiologic Studies Depression scale (CES-D) when people scored 16 or higher on this scale [15]. No other inclusion or exclusion criteria were defined for this study.

A total of 263 participants were randomized to one of the three conditions: Internet-based cognitive behavioral therapy (CBT, n = 88), Internet-based problem-solving therapy (PST, n = 88) and a waiting list control group with unrestricted access to usual care (WL, n = 87). Block randomization was used, with each block containing 9 allocations. An independent statistician conducted the randomization. The study protocol was approved by the Medical Ethics Committee of the VU University Medical Centre.

### Interventions

The problem-solving therapy-intervention was a Dutch adaptation of Self-Examination Therapy by Bowman [16]. Problem-solving therapy consisted of 3 steps. First, the participants made a list of the most important things in their lives. Second, they wrote down their current worries and problems and divided these into important and solvable problems, important but unsolvable problems, and unimportant problems. The core element of problem-solving therapy is to address the solvable important problems following a six-step procedure: describing the problem, brainstorming, choosing the best solution, making a plan for carrying out the solution, actually carrying out the solution, and evaluation. During the third step, the participants made a plan for the future in which they described how they would try to accomplish those things that matter most to them. The course took five weeks and consisted of one lesson a week.

The cognitive behavioral therapy intervention was based on the “Coping with Depression” course [17], Dutch version [18]. Cognitive behavioral therapy in this study included psycho-education and focused on skills such as relaxation, cognitive restructuring (including coping with worrying thoughts), social skills training, and behavioral activation, specifically increasing the number of pleasant activities. Cognitive behavioral therapy consisted of 8 lessons, 1 lesson a week followed by a booster session after 12 weeks.

Participants in both interventions were supported by a life coach via email during the intervention period. Support was directed at helping the participant to work through the intervention and not at developing a therapeutic relationship or giving advice on how to cope with depressive symptoms or other problems. The average time that therapists spent on each participant providing feedback and answering questions via email was estimated at 20 minutes per week.

### Outcome Measures

#### Generic and Clinical Outcome Measures

Participants received a baseline questionnaire prior to randomization and later at 5-, 8- and 12-weeks follow-up.

The central generic outcome was health-related quality of life derived by means of the EQ-5D of the EuroQol group. The EQ-5D consists of five health state dimensions (mobility, self-care, usual activity, pain/discomfort, and anxiety/depression) on which the respondent has to indicate his own health state [19]. An advantage of the EuroQol is that an overall utility score of quality of life can be obtained, which facilitates comparisons with other interventions and health states in other disease areas [20]. A utility refers to the value that individuals or society may place on a particular health state. This valuation is indicated by a number between 0 (the worst imaginable condition: death) and 1 (perfect health). This study used the Dutch tariff to value generic quality of life. The utility scores of the EQ-5D are used to calculate the quality-adjusted life-years (QALYs) gained during the follow-up period by weighing the length of time spent in a particular health condition by the utility [20].

In addition, our primary clinical outcome was severity of depressive symptoms as measured by the Center for Epidemiological Studies Depression (CES-D) scale─Dutch version [15]. The CES-D consists of 20 items and the total score varies between 0 and 60 with higher scores indicating greater depressive symptom severity. The cutoff of 16 and higher is commonly used to denote a clinically significant level of depressive symptoms [15].

#### Measuring Service Use and Costs

Costs are defined from the societal perspective and encompass (1) intervention costs, (2) costs related to health care uptake, (3) out of pocket expenses for the family and the patient, and (4) costs stemming from production losses due to work loss days and work cutback days. Costs were calculated in Euros (€ ) for the reference year 2007. Information on the participants’ use of health services and production losses was obtained with the Trimbos and Institute of Medical Technology Assessment Cost Questionnaire for Psychiatry [21]. Data on service use and costs were collected for 2 periods: the 4 weeks prior to randomization and the 12 weeks following randomization. The 3 cost categories distinguished were: direct medical costs, direct nonmedical costs, and indirect nonmedical costs.

Direct medical costs consisted of intervention costs and uptake of health care services, including costs of medication. The per-participant intervention costs were estimated at € 501 for cognitive behavioral therapy and € 338 for problem-solving therapy. The largest share of the intervention costs stemmed from receiving support during the interventions. Other costs comprised maintenance costs of the websites and participants’ time for working through the self-help material which was valued at € 8.83 per hour. Differences in intervention costs between the 2 interventions were mainly due to the variation in duration, with problem-solving therapy taking 5 weeks and cognitive behavioral therapy, 8 weeks. Health care services were costed by multiplying the number of health service units by their standard cost price [22] (see Table 1). The costs of antidepressants, anxiolytics, and hypnotics were calculated as the cost price per standard daily dose on a monthly basis as reported in the Pharmaceutical Compass [23]. Direct nonmedical costs consisted of costs for traveling and parking. These costs were valued at € 0.17 per kilometer and € 2.64 per hour parking time. To this we added the costs of the participants’ time spent in travel, waiting, and in treatment at € 8.83 per hour (see Table 1).

**Table 1:** Direct medical and direct nonmedical costs by health service type

		Direct Medical Costs^a^	Direct Nonmedical Costs^a,b^		
	Unit	Cost (€ )	Distance Traveled (km)	Patient’s Time (hrs)	Cost (€ )
General practitioner	Consultation	21.36	1.8	1	11.80
Regional mental health service	Contact	131.14	10	3	30.84
Private practice psychotherapist	Session	80.38	5	2	21.16
Psychotherapist hospital^c^	Consultation	76.08	7	3	30.31
Company doctor	Consultation	22.47	0	1	8.83
Social worker in company	Contact	50.62	7	3	30.31
Medical specialist	Consultation	103.64	7	2	21.48
Physiotherapist	Contact	24.06	1.8	2	20.63
Social worker	Contact	50.62	7	3	30.31
Consultation alcohol/drugs	Contact	131.14	10	3	30.84
Home care	Hour	32.47	0	0	0.00
Alternative care	Contact	47.00^d^	7	3	30.31
Self-help group	Session	8.83	7	3	30.31

^a^ Costs are in Euros for 2007.

^b^ Based on average distances (km) and travel+waiting+treatment times (hrs) for receiving treatment [22]

^c^ Valued as the mean of costs for a consultation in a general or mental health hospital

^d^ Costs for alternative care are variable. If unknown, the mean price of € 47 for various alternative forms of care was used.

Indirect nonmedical costs arise when production losses occur due to illness. Productivity costs were calculated according to the friction cost approach [22]. However, as our follow-up takes 12 weeks, the friction cost approach and the human capital method would produce the same results. Production losses can occur under 3 conditions. First, people can be absent from paid work due to sick leave (work loss days). To evaluate a lost day in a paid job, the average age- and gender-specific “friction costs” are used [22]. Second, production losses may occur when people are ill but continue to work with reduced efficiency (work cutback days). The number of work cutback days was estimated as the number of days actually worked when ill multiplied by a self-reported inefficiency score, which ranged between 0 (as efficient as when in good health) and 1 (totally inefficient). Third, people may also be too ill to perform domestic tasks. These costs were evaluated as the price of domestic help at € 8.83 per hour.

### Statistical Analyses

All analyses were performed in accordance with the intention-to-treat principle. The expectation maximization algorithm (EM) in Statistical Package for the Social Sciences (SPSS, version 17) (SPSS, Inc, Chicago, IL, USA) was used to estimate missing values. EM employs maximum likelihood estimation to replace missing values in the data set with estimates.

#### Analysis of Generic and Clinical Outcomes

Missing EQ-5D scores were imputed at 5-, 8- as well as 12-weeks follow-up using EM. The rationale behind imputing EQ-5D scores for all measurements is that QALYs were calculated for each different time period separately, that is, QALY1 was calculated from baseline to 5 weeks follow-up, QALY2, from 5 to 8 weeks, and QALY3, from 8 to 12 weeks. In this way, speed of change is taken into account as improvements often occurred in the early stages of the treatments. QALYs for the 3 time intervals were added over the total time span from baseline to the last follow-up at 12 weeks post baseline. QALYs were calculated by multiplying the utility values of the health state by the length of time spent in that health state.

Clinically significant change was determined with Jacobson’s and Truax’s algorithm for reliable and clinically significant change [24] while using the cutoff score of 16 on the CES-D as an indication of recovery. For a change to be clinically significant, a participant had to have recovered as well as had to have shown reliable improvement at 12-weeks follow-up.

#### Analysis of Costs and Economic Evaluation

Costs were determined at 5-, 8- and 12-weeks follow-up. Total cumulative costs over 12 weeks were determined by adding up the costs of each of the 3 time intervals with missing cost data imputed using the EM algorithm.

The economic evaluation consisted of a cost-utility analysis and a cost-effectiveness analysis. For both analyses, the incremental cost-effectiveness ratio (ICER) was calculated as (C_1_-C_0_)/(E_1_-E_0_), where C are costs and E is the effect and the experimental and comparator conditions are indexed with the 1 and 0 subscripts. The incremental cost-utility ratio will focus on the net costs per QALY gained. The cost-effectiveness ratio focuses on the net costs per reliable and clinically significantly improved case of depression. Nonparametric bootstrap resampling techniques (with 5000 replications) were used to take into account the stochastic uncertainty of the ICER estimates. In addition, the bootstrap analysis helped to obtain the median ICER and its 95% confidence interval from all 5000 simulated ICERS. The median and its confidence interval were based on the 50^th^, 2.5^th^, and 97.5^th^ percentile of the distribution of the 5000 bootstrapped ICERs. As the arrhythmic mean may not provide the best estimate for the ICER, we only present the median ICER.

The scatter plots of 5000 bootstrapped ICERs on the cost-effectiveness plane were generated. This helps to produce estimates of the probability that (1) better health is generated for more costs (northeast quadrant), (2) that the intervention is inferior relative to the control condition because less health is produced at additional costs (northwest quadrant), (3) that less health is generated for lower costs (southwest quadrant), and (4) that the intervention dominates because better outcomes are obtained for lower costs (southeast quadrant). Another way of illustrating the cost-effectiveness results is the cost-effectiveness acceptability curve. Such an acceptability curve represents the probability that the intervention is cost-effective relative to the comparator condition, given varying ceilings for the willingness to pay (WTP) for one quality-adjusted life-year or for one case of depression showing reliable and significant improvement.

#### Sensitivity Analyses

In the main analysis, maximum likelihood estimation was used to handle missing data. Due to high attrition, we repeated analyses using multiple regression to impute missing data.

## Results

### Participants

The average age of the 263 participants at baseline was 45 years (SD 12.1). Most participants were female (187/263, 71%) and the majority had higher vocational education/university (168/263, 64%) or intermediate vocational education/high school (72/263, 27%). Almost all participants were Dutch (243/263, 92%). The mean score of the participants on the CES-D was 31.7 (SD 7.5, median 31.0) at baseline. Mean utility score on the EQ-5D was 0.61 (SD 0.22). There were no statistically significant differences between the three groups with respect to the demographics, depressive symptoms, or quality of life scores at baseline [13]. At baseline, total per capita costs were € 846, € 893, and € 925 for the cognitive behavioral therapy, problem-solving therapy, and waiting list groups respectively. No significant differences were found in total costs between the three groups, *F*_2, 262_ = 0.10, *P* = .91.

### Clinical Outcomes

After 12 weeks, cognitive behavioral therapy and problem-solving therapy resulted in significantly higher quality of life scores and lower depression scores than waiting list placement. Full details of the main clinical outcomes are reported elsewhere [13].

The mean number of QALYs during the period of 12 weeks was 0.16 for cognitive behavioral therapy (95% CI 0.152 – 0.169), 0.16 for problem-solving therapy (95% CI 0.152-0.168), and 0.15 for wait-list (95% CI 0.142-0.159). No significant differences were found between groups, *F*_2, 262_ = 1.83, *P* = .16. Regarding clinically significant change, both cognitive behavioral therapy and problem-solving therapy tended to show more clinically significant change than waiting list placement after 12 weeks, χ^2^_2, 263_ = 5.10, *P* = .08. The number of participants showing clinically significant change was 25 (28.4%) for cognitive behavioral therapy, 23 (26.1%) for problem-solving therapy, and 13 (14.9%) for waiting list placement.

### Health Care Service Use

Cost data were available at baseline from 252 participants (cognitive behavioral therapy, n = 83; problem-solving therapy, n = 85; waiting list group, n = 84). At 12-weeks follow-up, 147 participants returned cost data (cognitive behavioral therapy, n = 45; problem-solving therapy, n = 40; wait-listed group; n = 62). For descriptive purposes, Table 2 presents the number of participants using health care services with productivity costs due to production losses, based on the sample of participants that returned cost data at baseline and at 12-weeks follow-up.

At baseline, more than half of the participants used some form of medication. This level of medication use continued into the follow-up period. Of all participants, 30% to 40% had contact with their general practitioner at both assessments, which happened to the same degree across all conditions. Reasons for visiting the general practitioner were, however, unknown. Frequently used services other than the general practitioner included visits to psychotherapists with a private practice, medical specialists, physiotherapists, and regional mental health services. At follow-up, visiting the private practice psychotherapist was reduced only in the problem-solving therapy group. The use of regional mental health services was reduced in both intervention groups.

With regard to production losses, a majority of the participants reported domestic costs at baseline with evident reductions reported by both intervention groups at follow-up. Reductions were also reported by both intervention groups in the number of participants that incurred costs due to work loss and work cutback in paid employment.

**Table 2:** Number and percent of participants using health care services and experiencing production losses

		At Baseline			At 12 weeks		
		CBT	PST	WL	CBT	PST	WL
Cost Item		n = 83	n = 85	n = 84	n = 45	n = 40	n = 62
**Health care services**		n (%)	n (%)	n (%)	n (%)	n (%)	n (%)
	Medication^a^	46 (56.1)	46 (54.1)	43 (51.2)	25 (55.6)	18 (45.0)	35 (56.5)
	General practitioner	37 (44.6)	26 (30.6)	34 (40.5)	18 (40.0)	14 (35.0)	22 (35.5)
	Regional mental health service	13 (15.7)	14 (16.5)	15 (17.9)	4 (8.9)	3 (7.5)	10 (16.1)
	Private practice psychotherapist	22 (26.5)	29 (34.1)	21 (25.0)	13 (28.9)	8 (20.0)	14 (22.6)
	Psychotherapist hospital	5 (6.1)	5 (5.9)	5 (6.0)	3 (6.7)	2 (5.0)	5 (8.1)
	Company doctor	6 (7.3)	10 (11.8)	6 (7.1)	3 (6.7)	1 (2.5)	6 (9.7)
	Social worker in company	4 (4.9)	2 (2.4)	2 (2.4)	0	0	1 (1.6)
	Medical specialist	19 (23.2)	15 (17.6)	13 (15.5)	10 (22.2)	9 (22.5)	9 (14.5)
	Physiotherapist	17 (20.7)	19 (22.4)	16 (19.0)	10 (22.2)	6 (15.0)	8 (12.9)
	Social worker	2 (2.4)	2 (2.4)	3 (3.6)	0	0	1 (1.6)
	Consultation for alcohol/drugs	0	0	1 (1.2)	0	0	1 (1.6)
	Home care	4 (4.9)	3 (3.5)	1 (1.2)	2 (4.4)	2 (5.0)	1 (1.6)
	Alternative care	10 (12.2)	12 (14.1)	17 (20.2)	3 (6.7)	4 (10.0)	7 (11.3)
	Self-help group	2 (2.4)	5 (5.9)	2 (2.4)	1 (2.2)	1 (2.5)	0
**Production losses**							
	Work loss	17 (20.7)	13 (15.3)	14 (17.7)	3 (6.7)	1 (2.5)	13 (21.0)
	Work cutback	31 (37.8)	31 (36.5)	37 (44.0)	22 (26.7)	8 (20.0)	28 (45.2)
	Domestic loss	59 (72.8)	69 (81.2)	68 (81.0)	23 (51.1)	16 (40.0)	49 (79.0)

^a^ All types of medication are included.

### Costs

Table 3 presents costs for the different cost categories based on imputed data. The majority of total costs were attributable to the indirect (productivity) costs. Indirect costs were, on average, lower by € 201 (€ 1900-€ 1701) and € 258 (€ 1900-€ 1642) for cognitive behavioral therapy and problem-solving therapy, respectively, relative to waiting list placement. The differences in total direct medical costs were largely due to the inclusion of intervention costs for both cognitive behavioral therapy and problem-solving therapy. Overall, the cognitive behavioral therapy group incurred extra costs of € 256 (€ 2814-€ 2558) compared with the waiting list group. Problem-solving therapy led to extra costs of € 147 (€ 2705-€ 2558) compared with waiting list placement. Total costs between groups were not significant, *F*_2, 262_ = 0.15, *P* = .86.

**Table 3:** Estimated per capita costs (in Euros) by condition during a 12-week period

Cost Item	Costs (€ ) for 2007		
	CBT	PST	WL
Intervention, total cost	501	338	-
Health care use, mean (SD)	441 (452)	513 (453)	472 (538)
Medication^a^, mean (SD)	17 (25)	15 (26)	18 (30)
Sum of direct medical costs^b^	958 (462)	888 (461)	490 (544)
Direct nonmedical costs	156 (156)	175 (162)	168 (203)
Indirect costs	1701 (2562)	1642 (2669)	1900 (3544)
Total costs^c^	2814 (2683)	2705 (2851)	2558 (3691)

^a^ Costs of antidepressants, anxiolytics, and hypnotics

^b^ Includes costs of intervention, health care use, and medication

^c^ Includes direct medical costs, direct nonmedical costs, and indirect costs

**Table 4:** Incremental cost-effectiveness ratio (ICER)

Comparison and Analysis		Cost-utility Analysis			Cost-effectiveness Analysis		
		Median ^a^	Lower Bound ^b^	Upper Bound ^b^	Median	Lower Bound	Upper Bound
**CBT vs WL**							
	Main analysis ^c^	22,609	-322,604	428,771	1817	-9203	18,369
	Sensitivity analysis ^d^	-52	-144,943	223,908	19	-3856	3688
**PST vs WL**							
	Main analysis	11,523	-285,551	401,101	1248	-18,719	23,742
	Sensitivity analysis	-22,779	-105,142	41,183	-2096	-10,796	3319
**PST vs CBT**							
	Main analysis	-21,888	-892,856	927,332	-36	-37,109	1,000,000
	Sensitivity analysis	-49,963	-737,794	653,203	3164	-22,844	152,180

^a^ Median ICER = 50th percentile of the 5000 bootstrap replications of the ICER.

^b^ Lower and upper bounds = 2.5^th^ and 95.5^th^ percentiles of the bootstrap distribution.

^c^ Main analysis was conducted with expectation-maximization imputation for missing observations.

^d^ Sensitivity analysis was based on regression imputation.

### Cost-utility: ICERs

**Figure 1:**
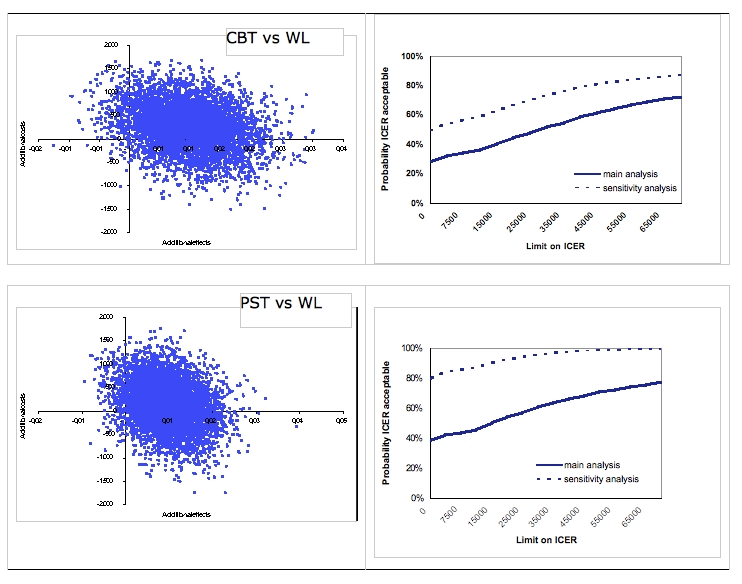
Distribution of bootstrapped incremental cost-effectiveness ratios (ICERs) (n = 5000) in the cost-effectiveness plane and ICER acceptability curve based on willingness to pay for one extra quality-adjusted life-year gained

Cost-utility analysis showed that the median incremental cost-effectiveness ratio for cognitive behavioral therapy versus waiting list placement resulted in € 22,609 per QALY gained. Hence, for each QALY gained by offering cognitive behavioral therapy instead of waiting, extra costs of € 22,609 are incurred. The left-hand panel of Figure 1 presents the distributions over the cost-effectiveness plane for the comparisons between the 2 active interventions and waiting list. Of the simulated ICERs, 28% are in the southeast quadrant, indicating a 28% probability that cognitive behavioral therapy is a better treatment because it generates more health effects for lower costs when compared with a waiting list control group. However, the majority of the simulated ICERs (67%) occur in the northeast quadrant, indicating that a health gain is produced, but at additional costs. Table 4 presents the median ICERs and their confidence intervals.

The median ICER for problem-solving therapy versus waiting list placement resulted in extra costs of € 11,523 per QALY gained by offering problem-solving therapy instead of waiting. In addition, there is a 58% probability that it is more effective in terms of QALYs at extra costs and there is a 37% probability that it is more effective at a lower cost.

Problem-solving therapy has a 35% probability of being the dominant treatment. And half of the ICERs are equally distributed over the northwest quadrant (24%) and the southwest quadrant (27%). The fact that the ICERs are almost equally divided over the four quadrants indicates no obvious preference for 1 of the 2 active interventions (Figure 3, left-hand).

### Cost-utility: Acceptability

The right-hand panel of Figure 1 presents the acceptability curves for the comparisons between the 2 active interventions and waiting list placement. Regarding the active interventions versus waiting list placement, there is a 28% and 38% probability respectively that cognitive behavioral therapy and problem-solving therapy are more cost-effective than waiting, if society places a zero value on one gained QALY. However, the probability of Internet-based therapy being more cost-effective increases when the willingness to pay for a QALY gained increases. To illustrate, with a willingness to pay ceiling of € 30,000 per gained QALY, then cognitive behavioral therapy and problem-solving therapy have a probability of 52% and 61% respectively of being more cost-effective compared with waiting.

### Cost-effectiveness: ICERs

**Figure 2:**
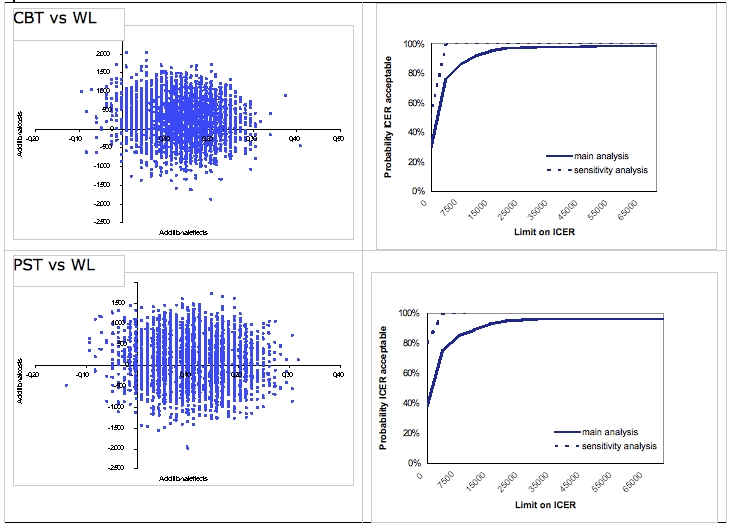
Distribution of bootstrapped incremental cost-effectiveness ratios (ICERs) (n = 5000) in the cost-effectiveness plane and ICER acceptability curve based on willingness to pay per clinically significant change in depressive symptoms

**Figure 3:**
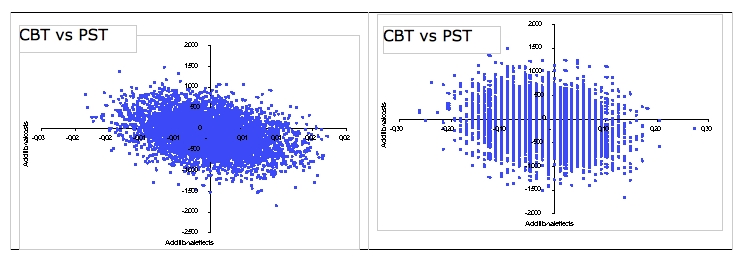
Distribution of bootstrapped incremental cost-effectiveness ratio (n = 5000) in the cost-effectiveness plane for quality-adjusted life-years (left-hand) and clinically significant change (right-hand).

In the cost-effectiveness analysis, the incremental cost-effectiveness ratio is expressed in terms of additional costs per clinically significant change in depressive symptom severity. The median ICER for cognitive behavioral therapy versus waiting list placement resulted in € 1817, indicating that by offering cognitive behavioral therapy instead of placing participants on a waiting list, extra costs of € 1817 are incurred for a health gain of one additional reliably improved participant. There is a 69% probability that a participant will change with cognitive behavioral therapy, but at additional costs. The distributions of the bootstrapped ICERs over the cost-effectiveness plane for the comparisons between the 2 active interventions and waiting list placement are presented in the left-hand panel of Figure 2. For problem-solving therapy versus waiting list placement, ICER is € 1248 per reliably improved participant. The probability that problem-solving therapy is more effective in terms of reduced depression severity but more costly than waiting is 60%. Comparing problem-solving therapy with cognitive behavioral therapy resulted in a median ICER of -36. As for cost-utility, the four quadrants each contain an almost equal percentage of the ICERs, meaning no evident preference for 1 of the 2 Internet-based treatments (Figure 3, right-hand).

### Cost-effectiveness Acceptability

The acceptability curve for cognitive behavioral therapy versus waiting list placement showed that with no willingness to pay for one reliably improved participant, there is a 30% probability that cognitive behavioral therapy is more cost-effective than waiting (Figure 2, right-hand panel). However, the probability rapidly increases when people are willing to pay more for clinically significant improvement. With a willingness to pay of € 5000 and € 10 000, cognitive behavioral therapy has a probability of 75% and 91% respectively of being more cost-effective compared with waiting list placement. For problem-solving therapy versus waiting list placement, the probability that problem-solving therapy is more cost-effective is 38% in the case of no willingness to pay. However, WTP of € 5000 and of € 10 000 result in probabilities of 75% and 89%.

### Sensitivity Analysis

Cost-utility and cost-effectiveness analyses were performed using multiple regression as an imputation method for missing observations. Regarding cost-utility, regression imputation led to better results for both interventions compared with the main analysis (where maximum likelihood estimation was used), the probabilities that the interventions result in better outcomes at lower costs than waiting list placement are 48% for cognitive behavioral therapy (was 28% when using maximum likelihood estimation) and 79% for problem-solving therapy (was 37% when using maximum likelihood estimation). For the comparison between the two active treatments, results are in favor of problem-solving therapy with a 72% probability that problem-solving therapy leads to better outcomes in terms of QALYs at lower costs. Cognitive behavioral therapy and problem-solving therapy have high probabilities (73% and 95% respectively) of being acceptable treatments with a WTP ceiling of € 30 000 (Figure 1, right-hand panel).

In the cost-effectiveness analyses, regression imputation also resulted in higher probabilities for the interventions being cost-effective compared with waiting list placement. In this case, the probabilities that the interventions will result in more clinically significant change at lower costs are 50% for cognitive behavioral therapy (was 30% when using maximum likelihood estimation) and 80% for problem-solving therapy (was 36% when using maximum likelihood estimation). And problem-solving therapy has a high probability (77%) of producing worse clinical outcomes at lower costs than cognitive behavioral therapy. With a WTP ceiling of € 5000, the probability of cognitive behavioral therapy being acceptable is 99% from a cost-effectiveness perspective (Figure 2, right-hand panel), while for problem-solving therapy this probability is 80% in the case of no willingness to pay.

## Discussion

### Main Findings

At first glance, cost-utility analyses produced conservative results regarding the efficiency of Internet-based treatment. With no willingness to pay for one extra quality-adjusted life-year, cognitive behavioral therapy and problem-solving therapy had a probability of only 28% and 38% of being more acceptable than waiting. But generally, people are willing to pay for one extra quality-adjusted life-year, although there is no consensus regarding the value of one QALY [25]. When the willingness to pay was raised to € 30 000, then the Internet-based interventions had a 52% and 61% probability of being the preferred option. With the same threshold of € 30,000, sensitivity analyses based on another way of imputing missing observations showed similar or higher probabilities (≥ 73%) of cognitive behavioral therapy and problem-solving therapy being acceptable treatments, thus attesting to the robustness of our findings. From a cost-effectiveness perspective, both treatments showed high probabilities of being more cost-effective compared with waiting even when society might place modest values on one clinically significant improved patient; cognitive behavioral therapy and problem-solving therapy showed a 91% and 89% probability respectively of being more cost-effective than waiting when society was assumed to have been prepared to pay € 10,000 for recovery from depression. Comparing both Internet-based treatments indicated no obvious preference for one of the treatments.

### Implications

Only a small number of depressed people are reached within traditional health care settings [26]. As Internet-based treatments are more accessible than traditional therapies, these treatments are assumed to be able to reach more people in need of treatment. Internet-based treatments for depression, mostly based on cognitive behavioral therapy, have been shown to be effective [12]. An important further step is to assess the cost-utility and cost-effectiveness of Internet-based treatments. Cost-utility analysis allows health care organizations and other stakeholders to compare the benefits of online treatment with other interventions across a range of disorders with which treatments for depression may be in competition for limited resources. The study of McCrone et al (2004) made a start by showing that computer-delivered cognitive behavioral therapy had a high likelihood of being cost-effective in primary care patients. Our study showed that two different Internet-based treatments with modest levels of therapist support were cost-effective in reducing depressive symptom severity in the general population, even in the conservative scenario that society had a limited willingness to pay for a reliably improved patient.

From a cost-utility point of view, the acceptability of the two treatments depends to a larger extent on what society is prepared to invest to achieve one quality-adjusted life-year. Differences in results between cost-utility and cost-effectiveness results can be explained by the nature of the outcomes. The quality-adjusted life-year is a measure of disease burden, including both the quality and the quantity of life lived, covering diverse health state dimensions, including mental health. The interventions were, however, directed at improving mental health, that is, reducing depression, and a depression-specific measure was used. Therefore, it cannot be expected that improvement in depression will result in across-the-board improvement in quality of life.

Besides, this study showed that a brief intervention based on problem-solving therapy seems to be a good alternative for Internet-based cognitive behavioral therapy in terms of cost-effectiveness. The generic nature of problem-solving therapy makes it suitable as a first step in a stepped care model. This would enable therapists to free up their limited resources and direct these to people presenting with more complex and severe symptomatology.

### Limitations

We acknowledge the following limitations of this study. First, we were faced with a high attrition rate. The use of maximum likelihood estimation to handle missing data might have introduced some bias. It is, however, a highly recommend method [27]. In a sensitivity analysis, outcomes and costs were, in addition, calculated using multiple regression as an imputation method for missing observations. Cost-effectiveness evaluations then became more favorable for both interventions. This implies that with high attrition rates, results partly depend on the imputation method used. For our data, it was understood that maximum likelihood estimation was the more conservative method; it was also the method used in the main analysis.

Second, the costs and effects were considered in the relatively short time span of 12 weeks. Accordingly, we do not know how the cost-effectiveness of Internet-based treatment is affected when a longer period is used, and this is a question that remains open for further research. Third, the study was not powered to detect statistically significant differences in outcomes and costs between the two Internet-based interventions. Therefore results regarding the comparison between the two active interventions should be considered explorative. Fourth, we used self-report for all measures. Self-report can be vulnerable to recall bias. The self-report of medication and care products for example, is often underestimated [28].

In light of these limitations, our findings should be interpreted with some caution.

## Abbreviations

CBT: cognitive behavioral therapy
CES-D: Center for Epidemiological Studies Depression scale
EM: expectation maximization
EQ-5D: EuroQol Questionnaire
ICER: Incremental cost-effectiveness ratio
PST: problem-solving therapy
QALY: quality-adjusted life-year
WL: waiting list control group
WTP: willingness to pay
